# The Role of the Valence, Arousing Properties and Subjective Significance of Subliminally Presented Words in Affective Priming

**DOI:** 10.1007/s10936-021-09815-x

**Published:** 2021-10-10

**Authors:** Kamil Imbir, Maciej Pastwa, Magdalena Walkowiak

**Affiliations:** Faculty of Psychology, University of Warsaw, 5/7 Stawki St., 00-183 Warsaw, Poland

**Keywords:** Affect misattribution, Valence, Arousal, Subjective significance, Subliminally presented words

## Abstract

In the verbal affective priming paradigm, the properties of a subliminally presented stimulus alter the interpretation of neutral target stimulus. In the experiment reported here, we tested the role of four factors (valence, origin, arousing properties and subjective significance) that determine the emotional reactions to words in affective priming. Subliminal masked presentation of words preceded the explicit task, which was assessment of neutral Quick Response code (QR code) stimuli. The QRs were codes for words representing personality traits. The results showed the effect of assimilation (negative words caused a negative interpretation, positive caused a positive interpretation) for words’ emotional valence and no effects for origin. Concerning arousal, we found a weak negative trend. In the case of subjective significance, a moderate positive trend was found. These results suggest that affective priming effects are susceptible not only to the valence of priming stimuli but also to activation factors.

## The Priming Phenomenon and Affective Priming

The nature of the boundaries of affect misattribution and priming effects is one of the most intriguing questions in psychology (Bussche et al., 2009). It appears that subliminal presentation of affective stimuli (both figurative and verbal) shapes the way we interpret subsequent neutral, external stimuli. In most cases, an “assimilation effect” is observed, with evaluation of a neutral target stimulus being aligned with the valence of the preceding subliminal emotional stimulus. This means that when a neutral object is preceded by a positive stimulus (e.g. a word), it is evaluated as more positive than it would be without the priming presentation, i.e. the positive affect from the first object was assimilated into the second one.

The best known subliminal affective priming procedure was introduced by Murphy and Zajonc (1993). Subjects were asked to appraise Chinese ideograms (the neutral stimuli), indicating whether they thought the ideograms had a positive or negative meaning or simply whether they liked them or not. The symbols were primed with emotional faces, which are a primal emotional signpost (Ekman, 1992). It turned out that items primed with faces expressing positive emotions were assessed as more positive and were liked better than those preceded by faces expressing negative emotions. Subliminal affective primes may influence the preference toward different items, including neutral faces (Donges et al., 2012; Li et al., 2008; Sweeny et al., 2009), pictures showing everyday situations (Hermans et al., 2003) or even a popular beverage (Winkielman et al., 2005). In a particularly interesting study, subjects evaluated the works of two famous abstract painters, Jackson Pollock and Hans Hartung (Flexas et al., 2013). The paintings were preceded by faces presenting positive or negative emotions (hereafter positive or negative faces). Paintings preceded by a smiling face were assessed more positively than those primed with negative faces.

In their original experiment, Murphy and Zajonc (1993) used pictures of faces as primes. It was quickly discovered that other emotionally charged stimuli, including emotional words, can be used in priming experiments (Abrams et al., 2002; Luo et al., 2004). A meta-analysis of studies using words as primes (Weingarten et al., 2016) concluded that they have a small but robust effect on behavior and that the observed effects obey the principles of value and goal satiation. In other words, subliminally presented words can, in fact, be perceived and processed and influence subsequent task performance. In affective priming or in affect misattribution (Payne et al., 2005), the direction of the effect induced by word primes is typically dependent on their emotional charge (Ferguson et al., 2005; Wentura et al., 2017; Zhang et al., 2012). Verbal stimuli and its emotional properties have been extensively explored in other procedures, such as the Emotional Stroop Task, while in the affective priming paradigm, the influence of words on reactions still needs exploring, especially when it comes to emotional factors other than valence (Burt, 2002). The aim of this experiment was to investigate the cognitive consequences of factors thought to be components of affective reactions to verbal stimuli, namely valence, origin, arousal and subjective significance (Imbir, 2016a) in affective priming.

## Factors Determining Emotional Reactions to Verbal Stimuli

The components of affective reactions to stimuli have been the subject of scientific attention since semantic differential studies (Osgood et al., 1957). The issue is important because defining affect dimensionally allows us to overcome one of the major problems in understanding how emotions shape mental processing (Kagan, 2007; Russell, 2003), by bypassing the need to approach each emotion separately. In simple terms, in everyday situations, we speak about emotions as discrete emotional experiences, using terminology that hinders understanding their complexity and their underlying mechanisms (Jarymowicz, 2012). Semantic differential studies (Osgood et al., 1957) identified valence, arousal and dominance/control as three aspects of emotional reactions to stimuli (Lang, 1980; Lang et al., 1997). Dominance/control appears to be highly correlated with valence (Imbir, 2016a; Moors et al., 2013; Warriner et al., 2013), so few studies have investigated how it affects cognition. Valence and arousal appear to be extremely useful in explaining the role of emotions in affective stimulus processing (Bayer & Schacht, 2014; Citron, 2012; Ortigue et al., 2004) and subsequent cognition (Imbir, 2016b; Payne et al., 2010; Pessoa, 2008; Zhang et al., 2012). Recognizing dimensions of affect allowed the development of theories of affect such as the constructionist view (Russell, 2003, 2009), stating that valence and arousal are the basic dimensions of “core affect”, the simplest kind of subjective affective experience. Recently a dual process approach was developed (Jarymowicz, 2012; Jarymowicz & Imbir, 2015), postulating that other important factors, for example, the origin of an affective state (automatic vs. reflective), or subjective significance (ascribing the activation specifically to reflective mechanisms of mind working) underlie the diversity of emotion (Imbir, 2015, 2016b). The core dimensions and the newly proposed dimensions can explain the diversity of subjective affective states.

### Valence and Origin of Affective Reactions

Valence is an intuitive dimension of emotions (Kagan, 2007; Kousta et al., 2009; Russell, 2003). The valence of a stimuli is determined by whether it elicits pleasure or displeasure (Lang, 1980) and by certain state of the body (Russell, 2003). The most commonly reported effects in affective priming studies were valence effects (Payne & Lundberg, 2014; Payne et al., 2005; Wentura et al., 2017). Assimilation occurs when appraisal of the target stimulus is congruent with features of the prime and contrast when appraisal of the target stimulus is incongruent with features of the priming stimulus (DeCoster & Claypool, 2004). In both cases, subliminally presented information has been processed and influenced the subjects’ behavior.

The origin (automatic vs. reflective) dimension was proposed recently (Imbir, 2015; Jarymowicz, 2012; Jarymowicz & Imbir, 2015) as a supplementary factor describing the complexity of mechanisms underlying emotional reaction formation. Emotions of automatic origin (hereafter automatic emotions) derive from automatic appraisal processes based on evolutionary fixed criteria, such as biological value (Damasio, 2010). Emotions of reflective origin (hereafter reflective emotions) derive from controlled appraisals involving verbalization (Strack & Deutsch, 2014) and based on evaluative standards (Jarymowicz, 2012; Reykowski, 1989), i.e. criteria for what is bad/avoidable or good/desirable. The origin of emotion measurement became possible with the creation of a self-assessment manikin scale (SAM) employing the heart vs. mind dichotomy (Imbir, 2015, 2016a). In metaphorical terms, the heart is commonly associated with inescapable, spontaneous reactions to stimuli (automatic reactions), whereas the mind (head) is associated with careful consideration and conscious decisions about how to behave. The origin SAM measures origin with a reliability comparable to that of other measures of affective reactions to stimuli (Imbir, 2015, 2016a) and has a stable pattern of correlations with other measures, so it can be used to operationalize the distinction between automatic and controlled mechanisms of emotional reaction formation.

Origin may be useful in understanding valence assimilation and contrast effects. It has been argued that situational factors determine whether assimilation or contrast is observed (Kobylińska & Karwowska, 2014). Assimilation of the valence of the priming stimulus is more probable if primes are presented subliminally, if subjects are cognitively distracted or have no prior information about the priming procedure, if the task does not engage reflective mechanisms and if the subject’s processing style tends not to be reflective (Kobylińska & Karwowska, 2014). Hence, reflective emotions, because they are based on propositional and reflective mechanisms (Strack & Deutsch, 2004), may lead to contrast effects in affective priming.

A single study investigated the influence of subliminally presented automatic or reflective emotional words on appraising neutral objects (Imbir & Jarymowicz, 2013). The authors presented Polish words, differing in origin and valence, for a period of 32 ms; after each word, a Japanese ideogram was presented. The participants had to evaluate whether the ideogram presented a negative or a positive trait. The results showed that automatic emotions led to an assimilation effect, with negative words evoking more negative appraisals than neutral ones, and positive words evoking more positive appraisals. Among the reflective words, the effect was different, with positive words leading to assimilation, while the negative ones evoked contrast; ideograms after both the negative and positive words were evaluated as more positive than in the neutral condition (Imbir & Jarymowicz, 2013). A similar study conducted recently, using a procedure of evaluating neutral stimuli after the optimal presentation of words differing with valence and origin, showed the assimilation effect to occur for words with automatic and reflective origins (Imbir & Pastwa, 2021).

The influence of the emotional origin of verbal stimuli on decisions not related to clearly evaluating the negative vs. positive traits has also been explored. The origin affects social cognition judgments, namely choosing whether an object represents warm or competent traits (Imbir, 2017b). In a task using purely ambiguous decisions of choosing between two unintelligible objects, the origin was found to influence reaction times (Imbir et al., 2018b; Imbir, 2017b) and neural correlates of processing (Imbir et al., 2018b). As for judging the words themselves, automatic originated words have been assessed as more emotional, while reflective originated words elicited longer reaction times (Imbir et al., 2019). In cognitive tasks, origin does not influence processing accuracy; however, automatic originated words have been shown to slow down reactions (Imbir, 2018). The results of many studies have also shown that the emotional origin of words influences the neural correlates of processing (Imbir et al., 2015; 2016a, 2016b, 2016c; 2017b).

### Arousal and Subjective Significance: Activation Underlying Emotional Reactions

Valence and origin are not sufficient to describe emotional reactions to words (Imbir, 2016a); activation-related factors (Imbir, 2016c; Russell, 2003) are also important. It has been postulated (Imbir, 2016b) that arousal and subjective significance are mechanisms for activating automatic and controlled processing (Epstein, 2003; Gawronski & Creighton, 2013; Strack & Deutsch, 2004), respectively. The theoretical framework for understanding the activation effects in affective priming may lead to the discovery of the role of so-called cognitive easiness of processing (Winkielman & Cacioppo, 2001). It appeared that more positive interpretations of neutral stimuli are easier to process (due to being more simplified in perceptual features or seen earlier and pre-processed), while more negative interpretations of stimuli are more difficult to process (due to their complexity, as well as first appearance). Activation makes processing easier. It appears that arousal activates simple processes directed at preserving life (such as the fight or flight reaction (Damasio, 2010)), but may disrupt more difficult cognitive processes (such as cognitive control; (Burt, 2002; Dresler et al., 2009; Imbir, 2016c)). The remaining issue is the identification of a mechanism that activates complex processing and disrupts simple processing. It has been proposed that subjective significance serves this purpose (Imbir, 2015, 2016c), Subjective significance is a rational evaluation of how important something is to one’s plans, expectations or priorities, and can also be measured with a SAM scale that is analogous to the arousal SAM scale (Imbir, 2015, 2016b). The reliability of measurement of the subjective significance of reactions to certain stimuli appears to be comparable to that of arousal and subjective significance, and arousal is moderately correlated. The cognitive easiness framework (Winkielman & Cacioppo, 2001) suggests that arousal would make the processing of the subsequent stimulus more difficult (neutral objects are abstract and geometrical in form), whereas subjective significance would make it easier; therefore, one would expect highly arousing stimuli to elicit a negative interpretation and highly subjectively significant stimuli a positive interpretation.

Affective priming was found to be susceptible to differences in the arousing properties of subliminally presented stimuli (Hinojosa et al., 2009; Thomas & LaBar, 2005; Zhang et al., 2012), although priming of discrimination in a lexical task was the subject of interest in these studies rather than the standard misattribution of affect to neutral stimuli effect. Nevertheless, the arousing properties of both the prime and target were found to influence the extent to which target stimuli were perceived as concrete or abstract (Thomas & LaBar, 2005), as well as response latencies and error rates for valence-incongruent picture (prime)-word (target) stimulus pairs and ratings of the pleasantness of target stimuli (Zhang et al., 2012).

Words also may be described as motivating action (there is some overlap between motivating action and being highly arousing) or being rather static in meaning (Imbir, 2016a). The action-inaction dimension seems to be important in understanding the influence of subliminal stimuli on subsequent tasks. Albarracin and Hart (2011) found that priming with action-related items improved subjects’ performance in the experimental task. Subliminal presentation of action words may reduce the time required to process a persuasive message (Albarracín & Handley, 2011) or influence behavior, e.g. increasing food intake (Albarracin et al., 2009). A very interesting finding is that highly arousing words may also influence personal goals, as action words motivate one to enter a dynamic state, whereas non-action words induce a desire to be in a static state (Albarracín et al., 2008). Items that elicit very low arousal may also influence behavior. Ginsberg et al. (2012) found that primes related to stereotypes of disability or old age reduced subjects’ motor performance.

Although subjective significance has not been investigated directly in studies using affective priming, there is some indirect evidence of the validity of the relationships postulated above. For example, Banse (1999) found that Chinese ideograms primed with the names of subjects’ friends or romantic partners, which we can assume were highly significant to them, were evaluated more positively than ideograms primed with other names. Subjectively significant stimuli may also influence cognitive operations other than simple likelihood assessment (Fitzsimons & Bargh, 2003). Priming subjects’ relationship representations (e.g. “Friend Study” vs. “Coworker Study”) produced goal-directed behavior (achievement, helping, understanding) in line with the previously assessed goal content of those representations. Koole and Coenen (2007) presented self-related words subliminally or supraliminally during an affective priming task (classification of words to differently valenced emotional categories) and, as they had hypothesized, found that self-concept-related primes influenced task performance. A general affective priming effect emerged with supraliminal self-primes but not subliminal self-primes (Koole & Coenen, 2007).

While subjective significance is a factor of emotional processing that was not studied extensively in the affective priming paradigm, in recent years, its influence on decisions and cognitive processing has been explored. Subjective significance of words presented before ambiguous stimuli influence interpreting them in terms of basic dimensions of social cognition; specifically, high subjective significance led to interpreting objects as competent rather than warm (Imbir, 2018). In another study, words with a high load of subjective significance were assessed as more emotional than the ones with low subjective significance (Imbir et al., 2018a). A high load of subjective significance also increases accuracy in a Lexical Decision Task (Imbir et al., 2020). High subjective significance also prolongs reaction times in tasks requiring decisions (Imbir et al., 2018a; Imbir, 2017a), while in purely cognitive tasks, high subjective significance shortens the reaction time (Imbir et al., 2017a, 2020). Subjective significance also influences neural correlates of processing in both decision and cognitive tasks (Imbir et al., 2017a, 2018a, 2020, 2021). Both arousal and subjective significance also tend to come into interaction when influencing reaction times in cognitive tasks, with a high load of subjective significance reducing the disturbance caused by high arousal (Imbir, 2017a).

## Aims and Predictions

The aim of the current experiment was to investigate how valence, origin, arousal and subjective significance influence affective reactions to verbal stimuli in form of emotion-laden words (Imbir, 2016a) in subliminal affective priming, using neutral QR codes as target stimuli. Emotion-laden words can be defined as words that do refer to emotions indirectly, in opposition to the emotion-label words ascribing the certain emotional states. Emotion-laden words elicit emotions due to their affective connotations stored in memories of experiences with objects (Pavlenko, 2008; Russell, 2003). We chose emotion-laden words due to the ecological validity. They do not trigger semantically emotions, but evoke emotional reactions to the stimulus itself. For that reason, we expected emotion-laden words to assure the validity of the results collected in the affective priming paradigm. We wanted to find out whether assimilation effects specific to valence dimension would be present for origin, arousal and subjective significance factors. We hypothesized (H1) that the valence of primes would be assimilated in subsequent cognitive processing, namely assessments of QR codes for personality traits, such that assessment would be more negative after subliminal presentation of negative words and more positive after presentation of positive words (Murphy & Zajonc, 1993; Payne et al., 2005). We also hypothesized (H2) that origin effects would interfere with valence effects, so that assimilation would only be observed in the case of stimuli that elicit an automatic emotional reaction. This would be in line with earlier studies, showing the origin to modulate valence effects in the affective priming paradigm (Imbir & Jarymowicz, 2013). Considering activational properties of neutrally valenced stimuli, we predicted distinct effects of arousal and subjective significance. Because arousal activates simple mental processes, we hypothesized that it would lead to negative outcomes in cognitive assessments (H3) and, hence, that highly arousing primes would result in negative interpretation of QR code stimuli and vice versa. Conversely, because subjective significance activates complex mental processes, we hypothesized that it would result in positive outcomes in cognitive assessments (H4) and, hence, that primes with high subjective significance would result in a positive interpretation of QR code stimuli and vice versa. Such effects for subjective significance would be consistent with previous findings, operationalizing this factor in other ways in priming tasks (Banse, 1999; Koole & Coenen, 2007). Effects for activation should account for the disruption (arousal) or facilitation (subjective significance) of complex processing required to give an answer to the target task (Imbir et al., 2017a, 2017b; Imbir, 2016c).

## Method

### Participants

A group of 54 people (38 women and 16 men) aged from 19 to 43 years (*M* = 25.1, *SD* = 4.1) participated in the study, which was presented as an investigation into the limits on intuition. The subjects were university students or had higher education qualifications in various fields, including social science, the natural sciences and the humanities. Participation in the study was voluntary and unpaid. The procedure was carried out in individual sessions in laboratory conditions. Polish was the mother tongue of all subjects. All subjects had normal or corrected-to-normal vision. Data from 6 people were excluded from analyses because they reported seeing words presented very briefly or could recall them (the consciousness test). Thus, the final sample consisted of 48 people (36 women and 12 men) aged from 20 to 43 years (*M* = 24.8, *SD* = 4.1).

Subjects provided verbal, informed consent to participate in the presence of at least one member of the research team and this was documented in a research diary. We did not collect any personal data from our subjects to ensure their anonymity. Before the start of the experimental procedure, subjects were informed that the study was investigating the limits of intuition and afterwards they were debriefed and informed about the actual aim and the use of subliminally presented words. This procedure was suggested by the bioethical committee of the Faculty of Psychology at the University of Warsaw. The design, experimental conditions and consent procedure for this study were approved by the same committee.

### Materials

Emotion-laden words were selected from a large set of Polish words for which affective norms have been determined empirically in Affective Norms for Polish Words-Reload (ANPW-R: Imbir, 2016a). The emotion-laden words constitutes a larger pool of stimuli than emotion-label words in Polish, what is especially important for the stimuli selection in orthogonal manipulations used. In the construction of the ANPW-R database, a group of 50 people rated the valence, origin, arousing properties and subjective significance of 4900 words. All the explored dimensions of word properties were assessed on a scale from 1 to 9, with descriptions differing for each scale. The descriptions for the scales were presented in a graphic, non-verbal way, as SAM scales (Imbir, 2016a). Valence was rated on a scale from 1 (word elicits negative feelings) to 9 (word elicits positive feelings), origin was rated on a scale from 1 (word elicits feelings of automatic origin) to 9 (word elicits feelings of reflective origin), arousal was rated on a scale from 1 (word elicits non-arousing feelings) to 9 (word elicits highly arousing feelings), subjective significance was rated on a scale from 1 (word elicits non-significant feelings) to 9 (word elicits highly significant feelings), and concreteness (treated in the present study as a control dimension) was rated on a scale from 1 (word represents abstract object) to 9 (word represents concrete object). Words classified as being (a) at least 1 *SD* below the mean, (b) within 0.5 *SD* of the mean or (c) at least 1 *SD* above the mean on each dimension were selected for experimental conditions in the present study. All the stimuli selected from the database were of medium ratings (within 0.5 *SD* of the mean) for control variables such as concreteness, frequency of appearance in the language and length. This allowed us to minimize the effect of the specific content of words and avoid experimenter biases. The affective norms database (Imbir, 2016a) allows researchers to select stimuli that differ systematically on the dimension of interest (e.g. valence) but do not differ on other dimensions (e.g. arousal, subjective significance, etc.).

### Valence and Origin Word List

A list of 135 words selected from the Imbir (2016a) database was divided into nine groups in a 3 (valence) × 3 (origin) model. We conducted six ANOVAs with the assessments of words on certain affective scales as dependent variables (valence, origin, concreteness, arousal, frequency of appearance in the Polish language, and length) and valence or origin group affiliation as independent variables in order to validate the selection of stimuli. Valence rating was affected by valence category *F* (2,126) = 607.44, *p* = 0.001, *η*^*2*^ = 0.91, but not origin category, *F* (2,126) = 1.88, *p* = 0.16, *η*^*2*^ = 0.03, and there was no interaction between valence category and origin category, *F* (4,126) = 2.09, *p* = 0.086, *η*^*2*^ = 0.062. Origin rating was affected by origin category, *F* (2,126) = 254.55, *p* = 0.001, *η*^*2*^ = 0.80, but not valence category, *F* (2,126) = 1.27, *p* = 0.28, *η*^*2*^ = 0.02, and there was no interaction between valence category and origin category, *F* (4,126) = 0.5, *p* = 0.74, *η*^*2*^ = 0.016.

Four additional dimensions describing the words were treated as controlled variables – arousal, concreteness, length of word and frequency of usage in Polish. Arousal rating was not affected by valence category, *F* (2,126) = 1.98, *p* = 0.14, *η*^*2*^ = 0.02, nor origin category, *F* (2,126) = 1.44, *p* = 0.24, *η*^*2*^ = 0.02, and there was no interaction between valence and origin categorization, *F* (4,126) = 0.5, *p* = 0.72, *η*^*2*^ = 0.016. Similarly ratings of concreteness were not related to valence category, *F* (2,126) = 1.19, *p* = 0.31, *η*^*2*^ = 0.02, nor origin category, *F* (2,126) = 0.4, *p* = 0.67, *η*^*2*^ = 0.006, and there was no interaction between these factors, *F* (4,126) = 0.12, *p* = 0.98, *η*^*2*^ = 0.004. Frequency of appearance in the Polish language (natural log-transformation of values from Kazojć, 2011) was not related to valence category, *F* (2,126) = 2.3, *p* = 0.11, *η*^*2*^ = 0.04, nor origin category, *F* (2,126) = 1.0, *p* = 0.37, *η*^*2*^ = 0.016, and there was no interaction between these factors, *F* (4,126) = 0.44, *p* = 0.78, *η*^*2*^ = 0.014. Finally, word length was also unrelated to valence category, *F* (2,126) = 2.01, *p* = 0.14, *η*^*2*^ = 0.03 and there was no interaction between valence category and origin category, *F* (4,126) = 0.82, *p* = 0.52, *η*^2^ = 0.025; however there was an effect of origin category, *F* (2,126) = 3.48, *p* = 0.034, *η*^*2*^ = 0.052. Post-hoc tests revealed a difference between words of automatic origin (*M* = 7.3; *SEM* = 0.3) and words with no particular origin, (*M* = 6.2; *SEM* = 0.3), *t* (132) = 2.62, *p* = 0.01; no other differences were detected. Mean ratings for all word groups on all dimensions are given in Table 1. The full list of words used is presented in "Appendix 1a".

**Table 1:** Means and standard deviations for nine groups of words organized by valence and origin

Level of origin		Level of valence						Total	
		Negative		Neutral		Positive			
		*M*	*SD*	*M*	*SD*	*M*	*SD*	M	*SD*
Automatic	Valence	3.50	.36	5.02	.56	6.71	.35	5.07	1.39
	Origin	4.45	.53	4.58	.37	4.33	.70	4.45	.55
	Arousal	4.37	.49	4.15	.55	4.28	.80	4.27	.62
	Concreteness	4.31	1.15	3.95	.74	4.48	1.20	4.24	1.05
	NoL	7.20	2.65	7.47	1.96	7.40	2.41	7.36	2.31
	Ln(freq)	5.21	1.91	5.65	2.03	5.73	2.28	5.53	2.04
Control (0)	Valence	3.37	.36	5.19	.54	6.38	.32	4.98	1.32
	Origin	5.41	.31	5.49	.30	5.36	.35	5.42	.32
	Arousal	4.15	.23	4.12	.67	4.04	.51	4.11	.49
	Concreteness	4.05	1.12	3.96	1.32	4.17	.74	4.06	1.06
	NoL	6.47	2.03	5.27	1.33	6.93	2.02	6.22	1.92
	Ln(freq)	5.48	2.28	5.97	1.27	6.61	2.02	6.02	1.92
Reflective	Valence	3.66	.35	5.30	.39	6.49	.40	5.15	1.23
	Origin	6.46	.30	6.63	.41	6.63	.56	6.57	.43
	Arousal	4.32	.49	3.93	.47	4.03	.36	4.10	.46
	Concreteness	4.17	1.13	4.09	1.17	4.41	1.07	4.22	1.11
	NoL	7.07	1.75	6.27	1.62	7.20	2.27	6.84	1.91
	Ln(freq)	5.42	1.37	6.53	1.79	6.01	1.22	5.99	1.52
Total	Valence	3.51	.37	5.17	.50	6.53	.38	5.07	1.31
	Origin	5.44	.92	5.57	.92	5.44	1.09	5.48	.97
	Arousal	4.28	.42	4.07	.56	4.12	0.58	4.16	.53
	Concreteness	4.18	1.11	4.00	1.08	4.35	1.01	4.18	1.07
	NoL	6.91	2.15	6.33	1.86	7.18	2.20	6.81	2.09
	Ln(freq)	5.37	1.85	6.05	1.72	6.12	1.89	5.85	1.84

### Arousal and Subjective Significance Word List

Another list of 135 words selected from the Imbir (2016a) database was divided into nine groups in a 3 (arousal) × 3 (subjective significance) model. ANOVAs of ratings were also carried out as described for the first list. Arousal ratings were related to arousal category, *F* (2,126) = 31.09, *p* = 0.001, *η*^*2*^ = 0.83, but not to the subjective significance category, *F* (2,126) = 0.88, *p* = 0.4, *η*^*2*^ = 0.01, and there was no interaction between arousal category and subjective significance category, *F* (4,126) = 0.5, *p* = 0.74, *η*^*2*^ = 0.016. Ratings of subjective significance were related to subjective significance category, *F* (2,126) = 35.62, *p* = 0.001, *η*^*2*^ = 0.81, but not arousal category, *F* (2,126) = 3.02, *p* = 0.053, *η*^*2*^ = 0.04, and there was no interaction between these factors, *F* (4,126) = 0.73, *p* = 0.6, *η*^*2*^ = 0.023. As the disproportion in *η*^*2*^ between arousal levels and subjective significance effects was large, we assumed that the manipulation words selection was accurate, regardless of the low *p*-value.

Four additional dimensions describing the words were treated as control variables, i.e. valence, concreteness, word length and frequency of usage. Valence rating was not related to arousal category, *F* (2,126) = 1.46, *p* = 0.23, *η*^*2*^ = 0.02, nor subjective significance category, *F* (2,126) = 1.89, *p* = 0.16, *η*^*2*^ = 0.03, and there was no interaction between these factors, *F* (4,126) = 0.23, *p* = 0.9, *η*^*2*^ = 0.007. Concreteness was another controlled characteristic and similarly, concreteness rating was not related to arousal category, *F* (2,126) = 0.03, *p* = 0.97, *η*^*2*^ = 0.001, nor subjective significance category, *F* (2,126) = 2.74, *p* = 0.07, *η*^*2*^ = 0.04, and there was no interaction between these factors, *F* (4,126) = 0.12, *p* = 0.9, *η*^*2*^ = 0.004. As in the previous list, frequency of usage (natural log transformation of the values given by Kazojć, 2011) was not related to arousal category, *F* (2,126) = 1.24, *p* = 0.29, *η*^*2*^ = 0.02, nor subjective significance category, *F* (2,126) = 2.99, *p* = 0.054, *η*^*2*^ = 0.05, and there was no interaction between these factors, *F* (4,126) = 0.19, *p* = 0.95, *η*^*2*^ = 0.006. Word length was also unrelated to arousal category, *F* (2,126) = 0.57, *p* = 0.57, *η*^*2*^ = 0.01, and subjective significance category, *F* (2,126) = 1.29, *p* = 0.28, *η*^*2*^ = 0.02, and there was no interaction between arousal and subjective significance categorization, *F* (4,126) = 0.19, *p* = 0.94, *η*^*2*^ = 0.006. The mean ratings of all word groups on all dimensions are given in Table 2. The full list of words used is presented in "Appendix 1b".

**Table 2:** Means and standard deviations for nine groups of words organized by arousal and subjective significance

Level of subjective significance		Level of arousal						Total	
		Low		Medium		High			
		*M*	(*SD*)	*M*	(*SD*)	*M*	(*SD*)	M	(*SD*)
Low	Arousal	3.20	.29	3.84	.27	4.79	.53	3.94	.76
	S. Significance	2.87	.33	2.96	.18	2.93	.60	2.92	.40
	Valence	5.25	.48	5.11	.44	5.01	.63	5.12	.52
	Concreteness	4.07	1.02	3.88	.73	3.91	.90	3.95	.87
	NoL	6.04	1.86	6.32	1.41	5.95	1.36	6.10	1.53
	Ln(freq)	5.73	2.09	6.20	1.32	6.07	2.02	6.00	1.81
Medium	Arousal	3.20	.16	3.85	.26	4.85	.35	3.97	.73
	S. Significance	3.56	.32	3.71	.22	3.74	.34	3.67	.30
	Valence	5.42	.47	5.38	.62	5.10	.66	5.30	.59
	Concreteness	3.92	.90	3.94	.90	3.98	.75	3.95	.83
	NoL	6.50	1.52	6.29	1.58	5.68	1.91	6.16	1.68
	Ln(freq)	6.27	2.09	6.13	1.68	6.87	2.45	6.42	2.07
High	Arousal	3.27	.27	3.85	.32	4.97	.33	4.03	.78
	S. Significance	4.55	.31	4.64	.40	4.88	.44	4.69	.41
	Valence	5.38	.36	5.41	.35	5.31	1.11	5.37	.69
	Concreteness	4.28	.75	4.33	1.00	4.37	.98	4.33	.89
	NoL	6.99	2.02	7.08	1.23	6.55	1.90	6.87	1.73
	Ln(freq)	6.47	2.13	6.67	1.95	6.87	2.00	6.67	1.99
Total	Arousal	3.22	.24	3.85	.28	4.87	.41	3.98	.75
	S. Significance	3.66	.77	3.77	.75	3.85	.93	3.76	.82
	Valence	5.35	.44	5.30	.49	5.14	.82	5.26	.61
	Concreteness	4.09	.89	4.05	.88	4.09	.89	4.08	.88
	NoL	6.51	1.81	6.56	1.43	6.06	1.74	6.38	1.67
	Ln(freq)	6.16	2.08	6.33	1.65	6.60	2.15	6.36	1.96

## Apparatus

A standard 15-inch laptop computer was used. The experimental protocol was designed using E-Prime 2.0 software. Subjects answered using the computer keyboard on which the keys that were active during the session were marked by stickers.

### Design

The study used two 3 × 3 within-subject designs: (a) valence (negative; neutral; positive) and origin (automatic; mixed; reflective) manipulation and (b) arousal (low; medium; high) and subjective significance (low; medium; high) manipulation. The dependent variable was the Quick Response code (QR code) assessment.

### Procedure

Brief instructions were displayed on the computer screen. Subjects were told that the study was an investigation in the limits of intuition and that their task would be to assess the QR codes, coding actual Polish words for various human traits. Participants were told that they would be asked to assess 270 QR codes. The task was to answer the question *What type of trait does this code represent?* using a five-point semantic differential scale ranging from 1—*negative* to 5—*positive*. The subjects were asked to respond as quickly as possible, by choosing one of the five keys marked on the keyboard, and to focus on the cross displayed in the center of the screen during the procedure.

**Fig. 1:**
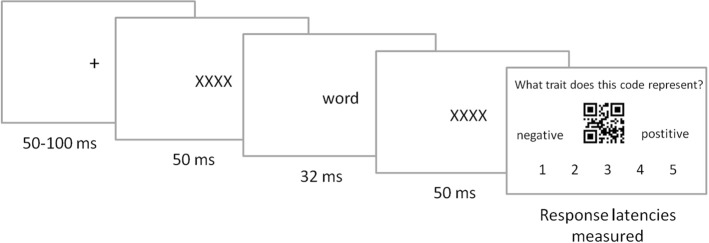
The structure of an affective priming trial: (1) a fixation point, (2) word masking, (3) presentation of an emotionally charged word, (4) word masking, and (5) QR code purported to represent a human trait, which has to be rated on a negative–positive scale

Both prime (emotionally charged words) and target (QR codes) stimuli were presented in random order; the lists for the two manipulations were presented in separate blocks, and the order of the blocks and the 135 words in each block was random. Thus the procedure consisted of 270 trials structured as follows: (1) presentation of a central fixation cross for a variable duration (random value between 50 and 100 ms), (2) the mask display (X letters string, their number dependent on the word length) lasting 50 ms, (3) 32 ms-presentation of word stimulus, (4) the mask display lasting 50 ms, (5) presentation of a neutral stimulus (QR code) and the response scale until a response was given. A single trial is presented in Fig. 1.

A brief awareness test was carried out after the experiment. Subjects were asked: (1) whether anything had caught their attention during the study, (2) if so, what, and (3) if they mentioned words, they were asked what the words were. After this, the actual aim of the study was revealed and the subjects’ questions were answered.

## Results

### Data Processing

Data were gathered from a total number of 14,580 trials involving 54 subjects (each performed 270 trials). Subject-wise exclusion of outliers in reaction times was applied. Response latencies more than 2.5 *SD* shorter (*n* = 106) or longer (*n* = 110) than the mean were excluded. Response latencies of less than 300 ms (*n* = 341) were also excluded. In total, 557 trials were excluded, which was about 3.8% of all trials. The remaining data for both lists were aggregated across subjects and conditions. The mean number of trials averaged for each condition was 14.43 (*SEM* = 0.11).

To test the hypotheses concerning priming effects of valence and origin, we conducted a repeated-measures ANOVA with two within-subjects variables: valence (negative; neutral; positive) and origin (automatic; mixed; reflective). Similarly, we tested the hypotheses concerning priming effects of arousal and subjective significance via a repeated-measures ANOVA with arousal (low; medium; high) and subjective significance (low; medium; high) as within-subject factors. In both ANOVAs, the dependent variables analyzed were (1) the answers given to a question concerning QR code meaning and (2) the reaction time of those answers. To control for right-skewed distributions of processing speed, we analyzed natural logarithm-transformed responses latencies, aggregated across conditions. Natural logarithm transformation is a standard procedure that allows parametric statistics to be used for reaction time data, which have a positively skewed distribution (Heathcote et al., 1991).

### Valence and Origin List

#### QR Code Interpretation

**Fig. 2:**
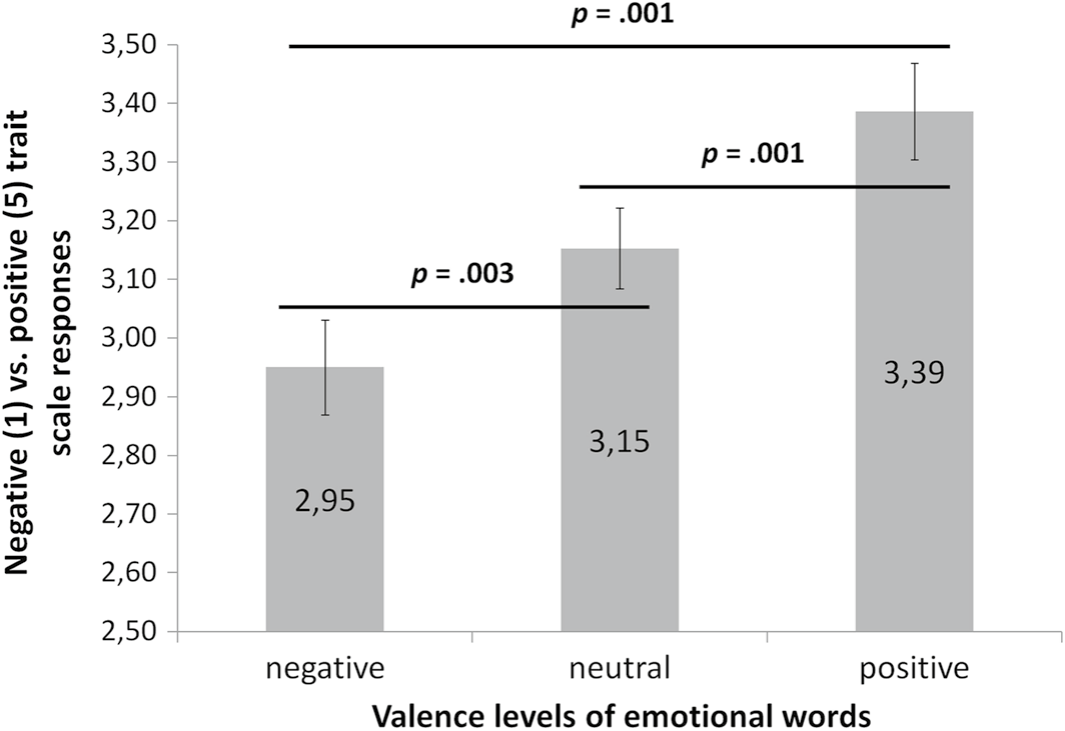
Mean assessments of QR codes on a 1 (negative) to 5 (positive) trait scale, grouped according to the valence category of the emotional prime used

There was a main effect of valence, *F* (1.26, 59.05) = 17.38, *p* = 0.001, *η*^*2*^ = 0.27. Simple contrast analysis showed differences between all valence levels. The QR code assessments for each valence level of prime were as follows: negative *M* = 2.95 (*SEM* = 0.08), neutral *M* = 3.15 (*SEM* = 0.07), positive *M* = 3.39 (*SEM* = 0.08). The QR codes were interpreted as representing more negative traits when negative words were used and more positive traits when positive words were used (*p* = 0.001). With neutral primes, the assessments were in between those and differed significantly from the negative (*p* = 0.003) and the positive conditions (*p* = 0.001). The pattern of results is shown in Fig. 2.

There was no effect of origin (*F* (2,46) = 0.02, *p* = 0.98, *η*^*2*^ = 0.001). The QR code assessments for each origin level of prime were as follows: automatic *M* = 3.17 (*SEM* = 0.07), mixed *M* = 3.16 (*SEM* = 0.07), reflective *M* = 3.17 (*SEM* = 0.06). There was no interaction between valence and origin level (*F* (4,44) = 2.39, *p* = 0.065, *η*^*2*^ = 0.18).

#### Response Latencies

With response latency as the dependent variable, there was no main effect of valence (*F* (2,46) = 0.5, *p* = 0.61, *η*^*2*^ = 0.02) or origin (*F* (2,46) = 0.42, *p* = 0.66, *η*^*2*^ = 0.02). There was also no interaction between valence and origin, *F* (4,44) = 0.19, *p* = 0.94, *η*^*2*^ = 0.02. The mean *ln* response latency was *M* = 7.2, *SEM* = 0.07 (raw data *M* = 1808 ms, *SEM* = 142 ms).

### Arousal and Subjective Significance List

#### QR Code Interpretation

**Fig. 3:**
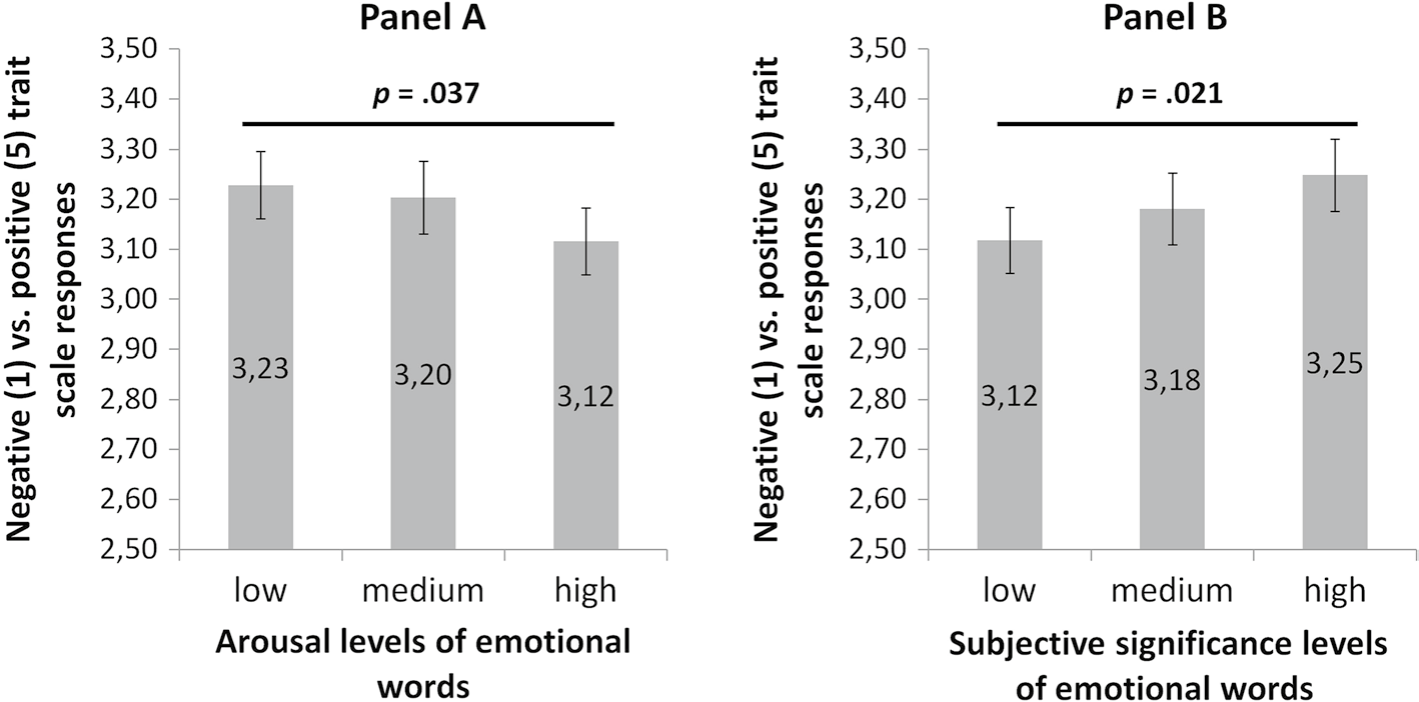
Mean assessments of QR codes on s on a 1 (negative) to 5 (positive) trait scale grouped by the arousal category (Panel A) and subjective significance category (Panel B) or the emotional prime used

There was a main effect of arousal, *F* (1.47, 68.85) = 4.44, *p* = 0.025, *η*^*2*^ = 0.09. The QR code assessments for each arousal level of prime were as follows: low *M* = 3.23 (*SEM* = 0.07), medium *M* = 3.2 (*SEM* = 0.07), high *M* = 3.12 (*SEM* = 0.07). Simple contrast analysis revealed that only assessments of targets primed with words eliciting low and high arousal were different (*p* = 0.037). The QR codes were interpreted as representing more positive traits when arousal was low, rather than high. The pattern of results is shown in Panel A in Fig. 3.

There was also a main effect of subjective significance, (*F* (2,46) = 4.21, *p* = 0.021, *η*^*2*^ = 0.155). The QR code assessments for each subjective significance level of prime were as follows: low *M* = 3.12 (*SEM* = 0.07), medium *M* = 3.18 (*SEM* = 0.07), high *M* = 3.25 (*SEM* = 0.07). Simple contrast analysis showed that only assessments of targets primed with words of high and low subjective significance were different (*p* = 0.021). The QR codes were interpreted as representing more positive traits when subjective significance was high, rather than low. The pattern of results is shown in Panel B in Fig. 3.

There was no interaction between arousal and subjective significance (*F* (4,44) = 1.27, *p* = 0.3, *η*^*2*^ = 0.1) with regard to QR code assessments.

#### Response Latencies

There was no main effect of arousal (*F* (2,46) = 0.24, *p* = 0.79, *η*^*2*^ = 0.01) or subjective significance (*F* (2,46) = 0.39, *p* = 0.68, *η*^*2*^ = 0.02) with response latency as the dependent variable. There was also no interaction between arousal and subjective significance, *F* (4,44) = 1.32, *p* = 0.28, *η*^*2*^ = 0.11. The mean *ln* reaction latency was *M* = 7.2, *SEM* = 0.07 (raw data: *M* = 1894 ms, *SEM* = 243 ms).

### Post-Hoc Power Analyses

We carried out post-hoc power analyses, using the observed sample effect size as an estimator of the population effect (Faul et al., 2007; Gelman & Carlin, 2014) to verify the adequacy of our sample size. We used the “ANOVA: repeated measures, between factors” option in the G Power Calculator (Faul et al., 2007). We found that the valence effect found for the valence and origin word list was highly powered, i.e. 1-beta error probability = 0.97. The subjective significance effect found for the arousal and subjective significance word list was on the verge of being highly powered, i.e. 1-beta error probability = 0.76, but the arousal effect was under-powered, with 1-beta error probability = 0.48.

## Discussion

We expected to find effects specific to each of the four dimensions investigated. Our hypotheses about valence, arousal and subjective significance were confirmed, but not the hypothesis about origin. The overall pattern of results suggests that it is not just the valence of primes that can influence how we interpret ambiguous, neutral target stimuli; the arousing properties and subjective significance of subliminally presented stimuli are also influential (cf. Antosz & Imbir, 2017; Imbir, 2017a, 2017b). Nevertheless, we have to acknowledge that in our experiment the valence effect was the strongest (*η*^*2*^ = 0.27), with the arousal effect (*η*^*2*^ = 0.09) and subjective significance (*η*^*2*^ = 0.155) effect being visibly weaker. The post-hoc power analyses revealed that although the valence and subjective significance effects had reasonable power (the sample was large enough to detect them), the arousal effect was underpowered (the sample was not large enough, and the analysis should be replicated in a bigger sample). This difference in effect sizes and power of the effects could be caused by the differences in ratings between groups of words on different dimensions. The differences in ratings from the original ANPW_R study (Imbir, 2016a) on the valence scale were the largest, with negative words at the level of *M* = 3.51 and positive words at the level of *M* = 6.53, while for other dimensions the differences were smaller; for example, the group of words with low arousal charge had a mean rating at *M* = 3.22 on the arousal scale, and the group of words with high arousal charge had a mean rating at *M* = 4.87. It is important to note that despite the discrepancies in differences between groups in absolute values, all the words were picked according to the distribution of values on the particular dimensions. Asking participants about valence charge leads to more extreme appraisals than asking for arousal or origin, which was taken into account when preparing the stimuli for experimental manipulation and verifying the selection with statistical analyses.

With respect to the valence and origin factorial manipulation, it should be noted that the valence effect we observed is consistent with the general pattern of results in the field, which exhibit the assimilation phenomenon (Monahan et al., 2000; Murphy & Zajonc, 1993; Payne et al., 2005). Negative words elicited more negative interpretations of QR codes than any other words, whereas positive words evoked more positive interpretations of QR codes than any other words. In other words, the valence of subliminally presented stimuli was the primary basis of assessments of the neutral object (Payne et al., 2010). When people do not have rational evidence on which to base an evaluation, they may be influenced by the affective state evoked by a stimulus. A duration of 32 ms and masked presentation of the word is standard procedure in verbal priming (Dijksterhuis, 2004; Klauer et al., 2007). With the first list of stimuli, we demonstrated that subjects’ assessments were susceptible to influence by the properties of the words used in the priming phase of the experiment. We expected valence effects to be modulated by the origin factor, but we found that this factor had no main effect and also did not interact with valence. It appears that origin does not influence the impact of priming stimuli on the interpretation of a target. This result is not consistent with an earlier experiment in which an interaction between origin and valence was observed (Imbir & Jarymowicz, 2013). In the earlier study, a different list of words was used (names of discrete emotional states or objects eliciting those emotions) and they were selected in a different way (on the basis of competent judge decisions, with no control over their arousing properties or concreteness), so the negative effects of the origin dimension can probably be accounted for by an arousal effect (higher arousal for automatic stimuli, lower for reflective stimuli). In other words, the stimuli in the earlier study may have differed systematically with respect to arousal, so the observed interaction may be artifactual. This interpretation is strengthened when the results with the second list used in the current experiment are considered.

With respect to the arousal and subjective significance factorial manipulation, we found that subliminal presentation of words that elicit low arousal made the interpretation of QR codes presented subsequently more positive, relative to subliminal presentation of highly arousing words. Arousal, related to the level of the organism’s vitality (Russell, 2003) or energy available in the organism (Osgood et al., 1957), is thought to activate simple processing (Epstein, 2003); therefore, high arousal impairs higher order processing, as captured in the Yerkes-Dodson laws (Imbir, 2016b; Teigen, 1994). The interpretation of ambiguous stimuli is an example of higher order processing. It is worth highlighting that our subjects were not asked to give a preference judgment, but to make a more complicated evaluation, to guess the meaning of stimuli—a variant of the procedure introduced by Murphy and Zajonc (1993). For that reason, one would expect highly arousing, neutral primes to disrupt processing, making it more difficult, and hence lead to more negative interpretations of neutral stimuli. Although we did not find differences in response latencies, the main effect of arousal on QR code assessments supports this argument; codes preceded by highly arousing stimuli were more likely to be interpreted as representing negative words than low arousing stimuli. We can conclude that this difference in interpretations of ambiguous stimuli was due to highly arousing primes increasing the effort required to process the target stimuli. It is also consistent with the results of experiments investigating the mechanisms underlying the mere exposure effect (Winkielman & Cacioppo, 2001), which have shown that differences in the cognitive ease of processing account for reactions to known objects being more positive than reactions to novel objects. The results of our experiment are not in line with some data from the lexical decision paradigm, showing that highly arousing neutral words enhance attention and speed up word processing (Recio et al., 2014). It is also likely that the type of task matters. Alternative explanations for the results we obtained should also be considered. For example, emotional words are overall less arousing than other emotional stimuli such as images or faces (Bayer & Schacht, 2014), so is possible that priming with highly arousing words results in more negative interpretations of subsequent neutral stimuli because the relationship between high arousal and extreme valence is stronger for negative primes than positive primes (Cacioppo, 2004; Võ et al., 2009).

We found that the manipulation of subjective significance produced the opposite pattern of differences to those observed with manipulation of arousal. Priming with words low in subjective significance elicited more negative QR code assessments than priming with words of high subjective significance. Subjective significance is a relatively new dimension (Imbir, 2015, 2016a, 2016b), ascribing the reflective form of an activation, and was introduced to explain why people engage in complex, effortful processing (Kahneman, 2011) instead of using heuristic thinking; they do so because they think the situation is worth the effort, because it is crucial, significant or constitutes a turning point with respect to their plans, aims, expectations or priorities. Subjective significance, due to being an activation for effortful processing, was found to reduce arousal-induced impairment in cognitive control in the emotional Stroop paradigm (; Imbir, 2016c), as indexed by response latency. We expected that, in the experiment presented here, priming with subjectively significant words would make the target task easier and, hence, result in more positive interpretations of the ambiguous QR codes. Response latency did not vary systematically with subjective significance, but the pattern of responses does suggest that subjective significance promotes positive interpretations of ambiguous stimuli. It should be noted, however, that there was no interaction between arousal and subjective significance, only an independent negative effect of arousal and positive effect of subjective significance. It is also worth noting that affective priming and the emotional Stroop task are quite different paradigms, measuring different aspects of mental processing, although both measure processes that are susceptible to the activation context (i.e. activating properties of a word); hence, one would expect some differences in the patterns of the results.

It is worth emphasizing that the QR codes used in this experiment appear to be good neutral stimuli. QR codes consist of black and white fields covering a standardized space and because they are used in everyday life they are, as a type, much more familiar to subjects than letters of a Far Eastern alphabet (Murphy & Zajonc, 1993) or even hexagram stimuli consisting only of black and white horizontal lines (Błaszczak & Imbir, 2012). Moreover, the instructions given for this experiment, stating that the QR stimuli represent words describing personality, was true. It was easier for subjects to believe that stimuli resembling codes they know from color magazines or food products (accessible with the use of a special application, allowing to decode them and read the information) really encode information. This enhances the ecological validity of affective priming paradigms. Another advantage is the ease with which QR codes can be created (numerous free software applications for doing so are available) and that a virtually unlimited number of distinct combinations of asymmetrical stimuli can be created (Monahan et al., 2000).

The main limitation of this study may be summarized as the use of two distinct lists of words created to compare the effects of the valence, arousing properties and subjective significance of priming stimuli. This decision was based on the desire for the strict separation of the factors to be investigated combined with matching of other potentially important factors (concreteness, frequency of usage and word length). The price paid for these stringent selection criteria was a reduction in the number of eligible stimuli. Nevertheless, in the arousal and subjective significance manipulation, the stimuli were matched for valence, which was neutral, so we aligned the main source of effects typically observed in affective priming. In light of the easiness of processing hypothesis (Winkielman & Cacioppo, 2001), it was also important to match word groups with respect to the frequency of usage and word length so that any observed effects could be attributed to the factors that had been deliberately manipulated. The strict control over non-target word properties is the main advantage of our study.

It has to be noted that we found only three significant effects, two of which, namely the effects of arousal and subjective significance, had rather small effect sizes. We did not observe any significant interactions between emotional factors, which could have been expected taking into consideration previous research (Imbir & Jarymowicz, 2013; Imbir, 2017a). A rather small group of participants constituting the final sample could be responsible for the lack of interaction effects, which had small effect sizes in the previous studies. We also did not observe effects regarding reaction times, which were common in previous studies exploring emotional factors manipulated in this experiment, both in decision and cognitive tasks (Imbir et al., 2017a, 2017b, 2018a, 2018b, 2019, 2020, 2021). The sample in the present study also had a large discrepancy between men and women, which not only could have affected the results, but also reduces the external validity of the study.

The results presented in this article contribute to the knowledge regarding implicit processing of emotional stimuli, confirming that valence is not the only emotional factor that may be processed subliminally (Citron et al., 2014; Cunningham et al., 2004). The fact that both the arousing value and the subjective significance of emotional stimuli may be processed without conscious acknowledgement of noticing the stimuli may be incorporated into therapeutic work. It is already a well-known fact that implicit factors such as micro-expressions can influence our mood and, as a consequence, mental stability (McDonald et al., 2018; Svetieva & Frank, 2016); therefore, it is possible that more factors other than the pleasant or unpleasant value of such implicit stimuli should be taken into account when analyzing its influence. The exploration of influence of four separate emotional factors on decision making, with clear results regarding the decisions themselves, also contributes to the field of research in subliminal affective priming paradigm, as most of the studies do not explore the factors of origin and subjective significance (Nava & Turati, 2020; Wang et al., 2021). The results of this study not only confirm the previous findings (Murphy & Zajonc, 1993; Winkielman & Cacioppo, 2001; Payne et al., 2005; Payne et al., 2010; Imbir & Jarymowicz, 2013), but also show new possible paths of research in this area.

To conclude, we have shown that the causes of affect misattribution are not limited to the valence of an affective state; affect misattribution also occurs when activating, but neutral words are presented subliminally. The pattern of results for arousal and subjective significance was in line with our hypotheses, which were derived from work highlighting the role of ease of processing in affect misattribution (Winkielman & Cacioppo, 2001). Our results also support the claim that arousal is a form of activation that promotes simplified cognitive processing (Epstein, 2003; Kagan, 2007; Kahneman, 2011) but disrupts more complex cognitive processes (Burt, 2002; Imbir et al., 2017a, 2017b). The effects of subjective significance obtained in the current study showed the opposite role of this factor in affective priming than would be expected in the theoretical model (Imbir, 2016b). In other words, the activation of complex processing may positively bias judgments of unknown objects, which would support the claim provided by the dual-processes model of emotion-cognition interactions, i.e. that subjective significance is in fact a reflective form of activation (Imbir, 2016b).

## Appendix 1a: Words used as experimental stimuli in Valence x Origin list

**Table:**

		Valence					
		Negative		Neutral		Positive	
		Polish	English	Polish	English	Polish	English
Origin	Automatic	Czkawka Szloch Łzy Uszczypnięcie Pijak Naiwniak Słabeusz Zmęczenie Hałas Plotka Grymas Gafa Usidlenie Smarkacz Zaślepienie	Hiccup Sob Tears Pinch Drunk Sucker Weakling Fatigue Noise Rumor Grimace Blunder Ensnaring Stripling Infatuation	Procesja Kościół Kuksaniec Tarot Loteria Westchnienie Jałmużna Błazen Mrowienie Pragnienie Obrzęd Wróżka Młodzież Łasuch Burza	Procession Church Nudge Tarot Lottery Sigh Alms Clown Tingling Desire Rite Fairy Youth Gourmand Storm	Zakochanie Passa Toast Powitanie Zapach Słodycz Pomoc Niemowlak Flirt Potomstwo Pozdrowienie Skarb Walentynka Podarunek Ferie	Infatuation Streak Toast Welcome Fragrance Sweetness Help Infant Flirt Offspring Greeting Treasure Valentine Gift Holiday
	Control	Wina Ciemnota Truchło Dół Ochłap Breja Paszkwil Kuternoga Reumatyzm Biedak Śpiączka Obtarcie Błąd Łachmany Wada	Fault Unacquaintance Carcass Pit Offal Slush Libel Lame Rheumatism Wretch Coma Sore Error Rags Drawback	Doping Chór Kłębek Telewizja Guru Wódka Unik Czara Smok Blef Żargon Głębia Farsa Grono Pisarz	Cheering Choir Hank Television Guru Vodka Dodge Goblet Dragon Bluff Jargon Depth Farce Bunch Writer	Przyjęcie Rejs Powiew Promocja Klimat Gość Brawa Kreskówka Melodia Wydarzenie Smak Południe Malarstwo Wyzwanie Obrońca	Party Cruise Waft Promotion Climate Guest Applause Cartoon Melody Event Taste South Painting Challenge Defender
	Reflective	Egzaminy Ignorancja Krata Minus Szpieg Koszty Podwładny Podatek Alimenty Odsetki Rząd Przemyt Recesja Bezrobocie Heretyk	Exams Ignorance Grating Minus Spy Costs Subordinate Tax Alimony Interest Government Smuggling Recession Unemployment Heretic	Szlachta Etykieta Sułtan Zadatki Prawo Prasa Stawka Raport Wojsko Interes Dyscyplina Wynik Weto Hodowla Kurs	Nobility Label Sultan Makings Right Press Bid Report Army Business Discipline Result Veto Breeding Course	Miliard Tolerancja Mistrz Patent Dobytek Absolwent Uczony Stypendium Szczyt Równowaga Oszczędności Płaca Satyra Lider Zysk	Billion Tolerance Master Patent Property Graduate Scholar Scholarship Peak Balance Savings Wages Satire Leader Profit

## Appendix 1b: Words used as experimental stimuli in Arousal x Subjective Significance list

**Table:**

		Arousal					
		Low		Medium		High	
		Polish	English	Polish	English	Polish	English
Subjective Significance	Low	Aspekt Echo Klan Mila Namiot Orbitowanie Pasmo Pauza Prasowanie Seria Sfinks Smuga Tenor Wersja Zero	Aspect Echo Clan Mile Tent Orbit Band Pause Ironing Series Sphinx Streak Tenor Version Zero	Akcent Bufor Gromada Hrabia Imam Loteria Marszałek Odcień Olbrzym Plemię Poganin Sabat Sługa Szlachta Wyłom	Accent Buffer Troop Count Imam Lottery Marshal Tint Giant Tribe Heathen Sabbath Servant Nobility Breach	Car Galop Harem Hazard Karykatura Koszary Kryminał Parada Salwa Smok Strzał Szaleństwo Wampir Wolt Zombie	Tsar Gallop Harem Gamble Pamphlet/caricature Barracks Thriller/jail Parade Salvo Dragon Shot Craze Vampire Volt Zombie
	Medium	Arka Fundusz Gamma Gleba Kawałek Kolor Laik Mgiełka Mieszkaniec Milczenie Połysk Północ Rzemiosło Saga Trasa	Ark Fund Gamma Soil Chunk Color Layman Haze Inhabitant Silence Shine North Craft Saga Route	Chrzest Grupa Interes Jazda Mrugnięcie Pokaz Posag Posiadacz Powieść Próba Swada Ulewa Waga Zadatki Zaułek	Baptism Group Business Ride Wink Show Dowry Possessor Novel Attempt Zest Downpour Weight Smack Alley	Bieganie Doping Duch Dziewica Labirynt Lesbijka Młodzież Mrok Mutacja Orgia Promieniowanie Sprint Turbo Wojsko Wyścig	Running Doping Spirit Virgin Maze Lesbian Youth Gloom Mutation Orgy Radiation Sprint Turbo Army Race
	High	Czyn Dokument Emerytura Godzina Istota Jednostka Krok Lekcja Osoba Poezja Singiel Woń Wykonywanie Zasada Zdanie	Deed Document Pension Hour Being Unit Step Lesson Person Poetry Single Odor Implementing Rule Sentence /sense	Biblia Budżet Dystans Firma Głębia Lekarstwo Nawyk Obserwowanie Praca Profesor Szczegół Uwaga Wpływ Wygląd Wymiana	Bible Budget Distance Business Profundity Medicine Habit Observation Labor Profesor Detail Note Influence Appearance Exchange	Alarm Buntownik Burza Ekstrawertyk Geniusz Majątek Maniak Ogień Płomień Poprawka Sesja Władza Wynik Wysiłek Zwycięzca	Alert/alarm Rebel Storm Extrovert Genius Fortune Maniac Fire Flame Amendment Session Authority Result Effort Winner
